# Comment on: Results from 237 extracorporeal membrane oxygenation runs with drowned patients

**DOI:** 10.1186/s13054-023-04624-1

**Published:** 2023-08-25

**Authors:** Romain Jouffroy, Benoît Vivien

**Affiliations:** 1 Service de Médecine Intensive Réanimation, Hôpital Universitaire Ambroise Paré, Assistance Publique - Hôpitaux de Paris, and Paris Saclay University, Paris, France; 2 SAMU de Paris, Service d’Anesthésie Réanimation, Hôpital Universitaire Necker - Enfants Malades, Assistance Publique - Hôpitaux de Paris, and Paris University, Paris, France

To the Editor,

We read with interest the recent research published in the Journal by Jasny et al. [1] reporting that survival among drowned adult and paediatric patients who received ECMO is lower than previously reported. Whereas the authors must be congratulated for this registry analysis, we believe that some points need to be emphasized in the interpretation of their results.

From a statistical point of view, it is surprising not to have compared patients cared for by cardiopulmonary resuscitation alone vs those with cardiopulmonary resuscitation plus ECMO. The latter are the most severe patients, consequently an adjustment including a prognostic score, i.e., SOFA or SAPS-2 [2, 3], would have been desirable in the multivariate analysis. Moreover, some potential confounders included in the multivariate logistic regression are censored, for example chronic pulmonary disease. As most patients received prehospital and in hospital cardiopulmonary resuscitation, OHCA outcome predictors could have been included in the multivariate logistic regression to take into account the CA characteristics impact on cardiopulmonary resuscitation results [4].

Finally, from a clinical point of view, the negative association observed between hospital mortality and stroke is surprising, since previous studies reported that ischemic and/or hemorrhagic stroke occurrence during ECMO is associated with an increased mortality rate, of almost 80% [5].

Beyond these limitations, we agree with Jasny et al. [1] that there is a great need for studies on potential ECMO benefits and its indications after adult and paediatric drowning is needed.

## Data Availability

Not applicable.
